# Cryo-EM structure of a light chain-derived amyloid fibril from a patient with systemic AL amyloidosis

**DOI:** 10.1038/s41467-019-09032-0

**Published:** 2019-03-20

**Authors:** Lynn Radamaker, Yin-Hsi Lin, Karthikeyan Annamalai, Stefanie Huhn, Ute Hegenbart, Stefan O. Schönland, Günter Fritz, Matthias Schmidt, Marcus Fändrich

**Affiliations:** 1 Institute of Protein Biochemistry, Ulm University, 89081 Ulm, Germany; 2 Medical Department V, Section of Multiple Myeloma, Heidelberg University Hospital, 69120 Heidelberg, Germany; 3 Medical Department V, Amyloidosis Center, Heidelberg University Hospital, 69120 Heidelberg, Germany; 4 Institute of Microbiology, University of Hohenheim, 70599 Stuttgart, Germany; 5 Institute for Neuropathology, Faculty of Medicine, University of Freiburg, 79106 Freiburg, Germany

## Abstract

Amyloid fibrils derived from antibody light chains are key pathogenic agents in systemic AL amyloidosis. They can be deposited in multiple organs but cardiac amyloid is the major risk factor of mortality. Here we report the structure of a λ1 AL amyloid fibril from an explanted human heart at a resolution of 3.3 Å which we determined using cryo-electron microscopy. The fibril core consists of a 91-residue segment presenting an all-beta fold with ten mutagenic changes compared to the germ line. The conformation differs substantially from natively folded light chains: a rotational switch around the intramolecular disulphide bond being the crucial structural rearrangement underlying fibril formation. Our structure provides insight into the mechanism of protein misfolding and the role of patient-specific mutations in pathogenicity.

## Abstract (web summary)

Systemic AL amyloidosis is caused by misfolding of immunoglobulin light chains and is one of the most frequently occurring forms of systemic amyloidosis. Here the authors present the 3.3 Å cryo-EM structure of a λ1 AL amyloid fibril that was isolated from an explanted human heart.

## Introduction

Antibodies are protein structures of utmost importance to human health. They underlie the humoral immune system and many top-selling biopharmaceutical agents; yet, they can be the basis of devastating human diseases with systemic AL amyloidosis (i.e. the amyloidosis caused by immunoglobulin light chains) being a particularly important one^1^. Moreover, antibodies or antibody fragments can misfold during biopharmaceutical production, leading to a great need to improve our understanding of the misfolding of these proteins^2^. Systemic AL amyloidosis belongs to the most common forms of systemic amyloidosis in industrialized countries^3^. In the USA it occurs with an incidence of ~9–14 patients per 1 million inhabitants^4^. The misfolding of immunoglobulin light chains (LCs), which are constituents of natural antibodies^5^, gives rise to the disease. Precondition is a clonal B cell disorder, such as a multiple myeloma, which elevates the concentration of one monoclonal LC in the serum.

The clinical and pathological disease manifestations are diverse and AL amyloid deposits can be found in different tissues and organs^6^. Especially important are those variants of the disease that are associated with cardiac amyloidosis. Cardiac involvement is a major cause of mortality^7^. Untreated patients show a median survival of 7 months after initial diagnosis^8^. The current treatment standard is to stop the production of LCs with chemotherapy directed against the underlying B cell clone. In case of advanced heart involvement, patients may additionally have to undergo a heart transplantation^9,10^, which provides access to large quantities of amyloid fibrils for research purposes.

Several studies demonstrate that the properties of the precursor LCs predispose patients to develop the disease or a specific disease variant. There is a preponderance of λ-LCs versus κ-LCs (λ:κ = 3:1) in patients with AL amyloidosis, while κ-LCs are more abundant (λ:κ = 1:2) in healthy individuals and in patients with multiple myeloma^11^. Mutations in LC domains can destabilize the protein and/or accelerate the fibrillation of model proteins in vitro^12–15^. The presence of the *IGLV1-44* germ line segment in the LCs correlates positively with cardiomyopathy, while the *IGLV6-57* germ line segment correlates positively with kidney involvement^7,16–18^.

Amyloid fibrils are much better established as pathogenic agents in systemic amyloidosis^19^ than in many neurodegenerative amyloid diseases that rather depend on toxic amyloid oligomers^20^. Although free LCs or LC oligomers can make pathological contributions to systemic AL amyloidosis^7^, cardiac pathology arises largely from massive amyloid fibril deposits that impair the natural ability of the heart to pump and to contract. So far, little is known about the structure of pathogenic LC aggregates. AL fibrils have generic structural characteristics of amyloid fibrils, such as a cross-β structure, a width of ~15 nm and a twisted fibril architecture leading to regularly spaced cross-overs^21–23^. A deeper understanding of the mechanism of LC misfolding and consequent disease pathology is hampered by a lack of detailed structural information.

In this study, we used electron cryo microscopy (cryo-EM) to determine the molecular structure of an amyloid fibril underlying the pathology in an AL patient with severe cardiac amyloidosis. The fibrils were previously shown to consist of a LC fragment that corresponds to residues Val3–Ser118 of a λ-LC^22^ and they match the size of other AL fibril proteins in cardiac amyloidosis^22,23^. The fibril protein is not glycosylated and encompasses mainly the variable light (V_L_) domain, which is typical for λ-AL amyloidosis^13,22,23^. Using negatively stained transmission electron microscopy (TEM), we recently demonstrated that the fibrils from this patient contain a dominant fibril morphology, exhibiting a width of 13.6 ± 0.9 nm, and a minor morphology with a width of 20.4 ± 0.4 nm^23^. The cryo-EM structure of this dominant fibril morphology, which we present here, is informative about the mechanism of LC misfolding and illuminates the role of patient-specific mutations for the development of amyloidosis.

## Results

### Structural rigidity of the extracted fibrils

Using a previously established protocol to extract amyloid fibrils from diseased tissue^22^, we obtained AL amyloid fibrils from heart muscle tissue of a patient who underwent a heart transplantation as a consequence of severe cardiac AL amyloidosis (Supplementary Table 1). The fibrils are derived from the germ line segments *IGLV1-44, IGLJ3*, and *IGLC2*, demonstrating that the fibrils are representative for a λ-subtype causing cardiac involvement. The dominant fibril morphology in the extract is relatively straight, indicating its resistance to bending deformations. Quantitatively, measurement of the fibril contour length and its end to end distance yielded values of 6.7 ± 0.5 μm for the persistence length and 2.78 ± 0.21 × 10^−26^ N m^2^ for the bending rigidity (Supplementary Figure 1).

### Fibril topology obtained by cryo-EM

**Fig. 1:**
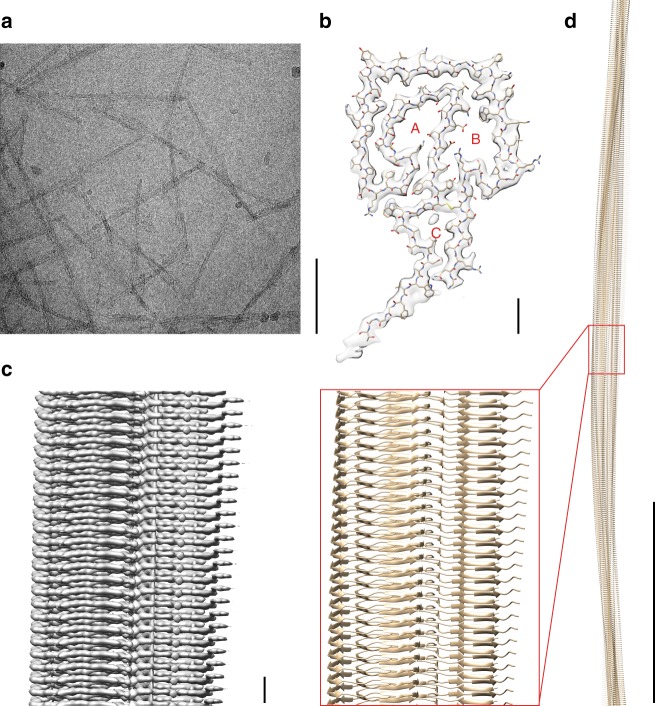
Cryo-EM structure of an amyloid fibril from systemic AL amyloidosis. **a** Raw cryo-EM image. Scale bar: 100 nm. **b** Cross-section of the reconstruction superimposed with a molecular model. Three internal cavities are labeled A–C. Scale bar: 1 nm. **c** Side view of the reconstructed density. Scale bar: 1 nm. **d** Side views of the molecular model. A segment corresponding to the reconstruction (**c**) is boxed. Scale bar: 50 nm

**Fig. 2:**
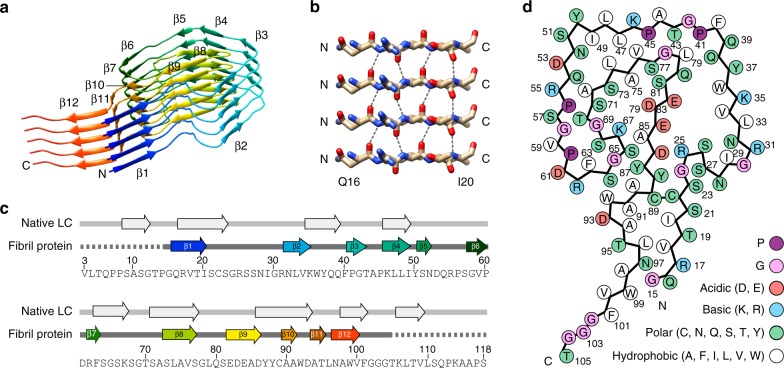
Fibril protein β-sheet structure. **a** Ribbon representation of a stack of five fibril proteins rainbow colored from N-terminus to C-terminus. **b** Close-up of the parallel cross-β sheet β1. **c** Position of the β-strands (arrows) in the fibril protein and in the natively folded LC as defined in PDB entry 1BJM^60^. Residue numbers refer to the native LC without signal sequence. The color of the β-strands in the fibril corresponds to panel **a**. Dotted gray line refers to the part of the protein that is disordered in the fibril. **d** Schematic representation of the fibril protein packing

Cryo-EM imaging of the extracted fibrils at 300 kV (Fig. 1a) allowed us to reconstruct the dominant fibril morphology at 3.3 Å resolution (Fig. 1b, Table 1) based on the 0.143 Fourier-shell correlation (FSC) criterion (Supplementary Figure 2). The two-dimensional (2D) class averages cover the entire fibril (Supplementary Figure 3). The reconstructed density shows a width of ~12 nm (Fig. 1b–d), in agreement with measurements from negatively stained samples^23^. The fibril consists of a single protein stack, termed here protofilament. It contains parallel cross-β sheets with intramolecular backbone hydrogen bonds (Fig. 2a, b). The fibril cross-section is asymmetrical (Fig. 1b), therefore a C1 symmetry was assumed during reconstruction. The fibril helix is left-hand twisted as confirmed by map-inversion and shows a pitch of ~300 nm as well as a polar topology (Fig. 1d).

**Table 1:** Cryo-EM data collection and image processing

Microscope	Titan Krios (Thermo Fisher Scientific)
Camera	K2 Summit (Gatan)
Acceleration voltage (kV)	300
Magnification	130,000
Defocus range (μm)	0.4–4.6
Dose rate (e^−^ pixel^−1^ s^−1^)	5.78
Number of movie frames	30
Exposure time (s)	6
Total electron dose (e^−^ Å^−2^)	32
Pixel size (Å)	1.041
Gatan imaging filter	20 eV
Mode	Counting mode
Box size (pixel)	320
Inter box distance (Å)	28.8
Number of extracted segments	119,395
Number of segments after 2D classification	62,250
Number of segments after 3D classification	32,677
Resolution, 0.143 FSC criterion (Å)	3.3
Map sharpening B-Factor (Å^2^)	119.6
Helical rise (Å)	4.8
Helical twist (°)	0.58
Symmetry imposed	C1

### Fold of the fibril protein

The three-dimensional (3D) cryo-EM map was fitted with a continuous polypeptide segment (Supplementary Table 2), corresponding to residues Gly15–Thr105 of the AL fibril protein (Fig. 2c, d). The protein N-terminal and C-terminal ends are juxtaposed in the structure and form a protruding stalk. The remaining part of the protein roughly outlines the shape of a ram head (Supplementary Figure 4). Head region and stalk lie on either side of an intramolecular disulfide bond that is formed between residues Cys22 and Cys89 (Fig. 2c, see below). The N-terminal and C-terminal ends of the stalk are surrounded by diffuse density (Supplementary Figure 4), indicating structural disorder of the first and last 12 residues (Fig. 2c).

The fibril protein belongs to the all-beta class of protein folds, consisting of 12 β-strands (β1–β12). The strands vary in length from two to eight residues. The folded structure shows several non-local contacts, such as between segments Gln16–Val18 and Asn97–Trp99, Cys22–Arg25 and Glu84–Cys89, Trp36–Gly58 and Thr70–Gln80, as well as Arg62–Lys67 and Asp83–Trp92. The polypeptide chain changes height by 8.2 Å along the fibril main axis (Supplementary Figure 5a), interdigitating the fibril proteins in the direction of the main axis. The intermolecular interactions in the fibril rarely extend beyond the next molecular layer. For example, strand β4 from layer *i* is in contact with strand β8 in layers *i* and *i* + 1 (Supplementary Figure 5b). Residue Arg25 interacts with residues Glu84 and Asp86 from layer *i* + 1 (Supplementary Figure 5c). The height change produces different tip structures at the two fibril ends (Supplementary Figure 5d), resembling other cross-β fibrils^24–28^. It was suggested that this fact may give rise to different mechanisms/kinetics of fibril outgrowth^26,29^.

The protein fold encloses three major cavities, labeled A–C (Fig. 1b). Cavities A and B are hydrophilic and occur within the head region. They are lined with many polar and ionic amino acid side chains, suggesting the presence of water. Cavity C is hydrophobic and located within the stalk region. This cavity is lined with hydrophobic side chains and the intramolecular disulfide bond. It also contains a small density that cannot be assigned to the polypeptide chain (Fig. 1b), indicating the presence of a molecular inclusion of low polarity.

### Location of sequence elements in the fibril protein

**Fig. 3:**
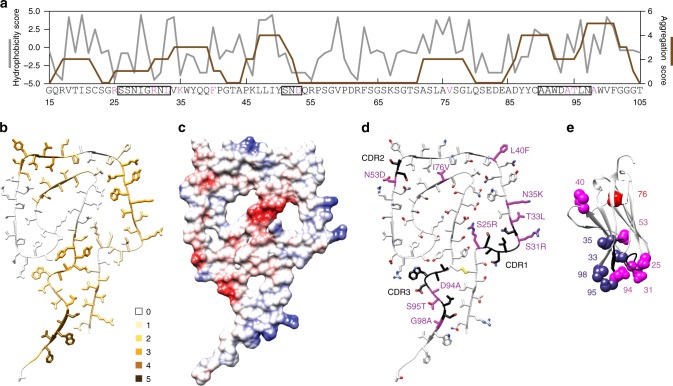
Location of specific sequence elements in the structure. **a** Hydrophobicity (gray) and aggregation score (brown) of the ordered part of the fibril protein. Magenta letters: mutations compared to the *IGLV1-44* germ line segment. Boxes: CDRs. Residue numbers refer to the native LC without signal sequence. **b** Fibril protein showing the residue-specific aggregation score (0–5). **c** Electrostatic surface representation of the fibril protein. **d** Fibril protein with CDRs (black) and mutations (magenta) highlighted. **e** Ribbon diagram of a native V_L_ domain (PDB entry 1BJM)^60^ showing residues 3–113. CDRs are colored black; mutations are colored in red if they affect the core (residue 76), purple (surface residues with potential relevance for domain–domain interactions), or magenta (other surface residues)

The most aggregation-prone segments of the protein exist at residues Asn97–Phe101, Lys46–Tyr50, Tyr87–Ala91, and Val34–Gln39 (Fig. 3a, Supplementary Table 3), similar to other λ1-LCs^30^. These segments do not correspond well with the β-strands in the fibril (Fig. 2a, c). Three segments are solvent-exposed, while residues Tyr87–Ala91 are buried (Fig. 3b). The fibril protein lacks a typical hydrophobic core (Fig. 2d). One of its most central structural elements is stand β9, which contains a highly acidic motif (Fig. 2d, Fig. 3c). The charges of this segment are only partially compensated by buried residues of the opposite charge (Arg25 and Lys73) (Fig. 2d). The complementarity determining regions (CDRs) are located on the fibril surface (Fig. 3d) and there are 10 mutations compared with the amino acid sequence encoded by the *IGLV1-44* germ line segment (Fig. 3d).

Three of these mutations add a surface charge to the fibril (Ser31Arg, Asn35Lys, Asn53Asp) (Fig. 2d). One mutation removes a charge from the non-polar cavity C (Asp94Ala). Ser25Arg inserts a basic residue into the polar cavity B, where it helps to compensate the charges of Glu84 and Asp86 and to interdigitate two molecular layers of the fibril Supplementary Figure 5c). Gly98Ala occurs in the highly aggregation-prone segment at residues Asn97–Phe101 (Fig. 3a). Thr33Leu, Leu40Phe, Ile76Val, and Ser95Thr have no obvious structural effect on the fibril. None of the replacements is clearly unfavorable to the fibril structure. No mutational change occurs within the *IGLJ3* and *IGLC2* germ line segments, nor within residues Gln1–Thr13, a previously described mutational hot spot of some amyloidogenic λ-LCs^31^. This segment is conformationally disordered or missing in the fibril (Supplementary Figure 4), which implies that at least for some patients this segment is not relevant to fibril formation. Indeed, mutational changes to the N-terminus were found to be more relevant to a λ6-LC-derived V_L_ domain, which is destabilized upon mutation^32^, and λ6-LCs may form fibrils with an ordered N-terminus^29,33^.

### Comparison with native LC conformations

The majority of the mutations occur at surface positions in the globular V_L_ domain (Fig. 3e). Four of these changes (Thr33Leu, Asn35Lys, Ser95Thr, Gly98Ala) may potentially impact the interactions with other immunoglobulin domains. Only Ile76Val affects a buried residue. This mutation removes a methylene group from the protein core, which typically destabilizes a protein by 6 kJ/mol^34^. Taken together, we find one out of 10 mutations in this patient to be unfavorable to the native state, while two to six changes make the LC more compatible with the fibril structure than the germ line segment.

**Fig. 4:**
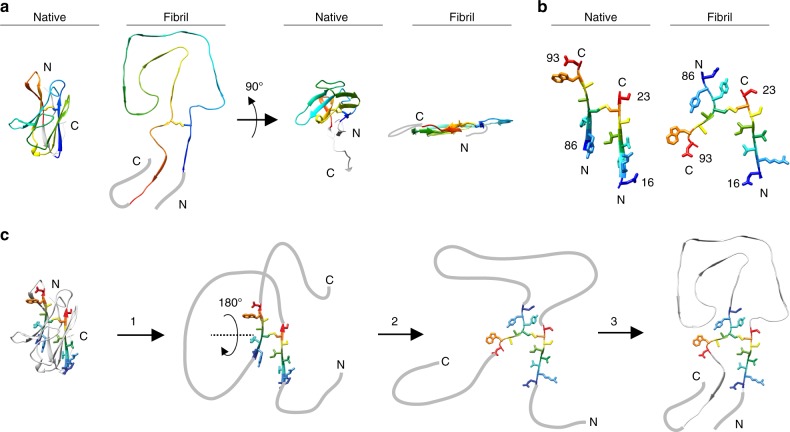
Comparison of the fibril structure and the native V_L_ domain fold. **a** Comparison of the native V_L_ domain fold (PDB entry 1BJM)^60^ with the fibril state. Residues 15–105 are shown in rainbow color. The native conformation is truncated at residue 118, corresponding to our fibril protein. The diffuse N-terminal and C-terminal tails of the fibril structure are schematically added with a gray line. **b** Part of the native structure and of the fibril state showing the conformational switch of segments 86–93 and 16–23 relative to one another around the protein disulfide bond. **c** Schematic representation of the hypothetical misfolding reaction consisting of an unfolding reaction (1), the rotational switch (2) and the assembly into the fibril structure (3)

The fibril structure is profoundly different from a natively folded V_L_ domain. Both protein states contain a high β-sheet content, but there are differences in the number and position of the β-strands within the sequence (Fig. 2c). The fibril conformation is more extended and flattened compared to the native state (Fig. 4a), enabling the polypeptide chain to form one molecular fibril layer. A particularly substantial structural rearrangement happens in the region around the disulfide bond which cross-links the N-terminal and C-terminal segments of the protein. These segments show a parallel N to C orientation in the native state and an antiparallel orientation in the fibril (Fig. 4b). Therefore, misfolding induces a 180° rotational switch of one segment relative to the other around the disulfide bond, placing the stalk on one side of the disulfide bond and the head region on the other (Fig. 4c). These stark structural differences between the native and misfolded state are consistent with our previous observation that AL amyloid fibrils and refolded fibril proteins differ in several structural features, such as their infrared spectral characteristics and their affinity for the amyloid-binding dyes Thioflavin T and Congo red^23^.

## Discussion

In this study, we have analyzed the molecular structure of an amyloid fibril that was purified from patient tissue and is therefore directly relevant to disease. Interestingly, we previously found that the fibril protein constituting this fibril is able to form amyloid-like fibrils in vitro that possess a different morphology, and possibly also a different protofilament substructure, than the bona fide pathogenic aggregates studied here^23^. These observations indicate that it is essential to investigate patient-derived rather than in vitro formed fibrils when scrutinizing the molecular basis of a protein misfolding disease.

The reconstructed fibril has an elongated and rigid structure, consisting of a single protofilament. Values measured for the bending rigidity of the AL fibrils (2.78 ± 0.21 × 10^−26^ N m^2^) correspond to previously reported values, which were on the order of 10^−28^–10^−25^ N m^2^ for different amyloid-like fibrils^35^. The stiffness of the fibrils could explain why amyloid fibril deposits impair the natural function of the heart. Cryo-EM and reconstruction of the 3D map enabled us to reveal the molecular structure underlying these effects. We could show that residues Gly15–Thr105 adopt a stable conformation in the fibril, while the first and last 12 amino acid residues are not resolved in our structure and are conformationally disordered (Supplementary Figure 4). The fibril proteins are interdigitated along the main axis of the fibril, providing resilience to mechanical stress (Supplementary Figure 1). The fold of the AL fibril protein is novel and differs from previously published fibril protein structures. It consists of a head and stalk region and encompasses one non-polar and two polar cavities (Figs. 1b, 2d).

The single-protofilament architecture of this AL amyloid fibril contrasts with the majority of previously described cross-β fibrils, which consist of multiple protofilaments^24,26–28,36^. However, we cannot exclude the formation of multi-protofilament AL amyloid fibrils, as our sample contains a second fibril morphology that is thicker than the presently studied one^23^. Furthermore, the presently investigated fibril contains surface-exposed charge pairs, for example at residues Asp53 and Arg55, which were previously identified as protofilament–protofilament interaction sites in murine AA amyloid fibrils^28^. However, the majority of the fibrils in the AL fibril sample does not contain multiple protofilaments, indicating that the assembly into higher-order structures is unfavorable for this fibril protein.

The protein fold provides insight into the mechanism of LC misfolding. It does not support mechanisms that assume of the initial formation of fibril segments consisting of dimeric proteins or peptides^13,37,38^. Nor is there evidence for an assembly of domain-swapped molecules, consistent with other studies^37,39^. The substantial conformational differences between the fibril structure and the native state imply instead that the native conformation must be largely, if not entirely, unfolded to allow fibril formation to occur (Fig. 4c). In particular, we identified a rotational switch of the polypeptide chain around the disulfide bond that is only possible if most of the native strand–strand interactions are lost. Unfolding and the rotational switch are crucial for fibril formation as unfolding is the prerequisite for the rotational switch, which in turn represents the basis for the formation of a flat protein structure that lacks chain crossings (Fig. 4c). This conformation is then able to associate into the intermolecular hydrogen bond network of a cross-β sheet.

As the disulfide bond occurs at the same position in the fibril as in the native V_L_ domain, previous research suggested that the misfolding of the LC occurs under oxidizing conditions and maintains the cysteine disulfide bond^23^. Our structure lends further support to this view, as we reveal a number of notable imperfections in the fibril packing. There are three major internal cavities (Fig. 1b) and the protein is packed inside-out or outside-in. A highly acidic segment of low aggregation propensity is buried in the core and only partially compensated by basic charges, whereas segments that are much more hydrophobic and aggregation-prone are exposed to the solvent (Fig. 3b, c). The situation looks drastically different in systemic AA amyloidosis, where the most aggregation-prone and hydrophobic segments are buried in the fibril core and where buried acidic residues are compensated by an equal number of buried basic residues^28^. AA amyloid fibrils, which contain no disulfide bond, accomplish a tighter packing than AL amyloid fibrils and form much smaller internal cavities.

The detailed information provided by our structure helps to explain the effects of mutational variants in systemic AL amyloidosis. Several mutational changes have a beneficial effect on the fibril structure (Fig. 3d), while only one mutation is clearly unfavorable to the native protein conformation (Fig. 3e). The analyzed fibril is representative for amyloid fibrils from cardiac AL amyloidosis as it is derived from an *IGLV1-44* germ line segment, the major germ line segment leading to heart involvement^7,16–18^. The mass of the fibril protein corresponds to that of other AL fibril proteins and consists mainly of a V_L_ domain^22,23^. The observed fibril morphology resembles fibril morphologies from other AL patients with cardiomyopathy^22,23^, despite clear patient-specific differences. However, systemic AL amyloidosis is an extraordinarily variable disease and other LC subtypes may be associated with different structural properties, particularly at the protein N-terminus, as was indicated recently for fibrils derived from λ6-LCs^29,33^.

The current data from cryo-EM sheds light onto the misfolding of proteins and their pathogenicity, representing a solid basis for further investigation of molecular mechanisms underlying human pathology, for example by in vitro aggregation studies. Detailed knowledge of the molecular structure of pathogenic protein states may lead to the development of novel ligands which recognize these structures and form the basis of new detection methods or therapeutic strategies. However, due to the heterogeneity of systemic AL amyloidosis further work will be necessary to dissect the structural characteristics of fibrils from different groups of patients and to identify common structural themes between different cohorts of patients as well as systematic variations.

## Methods

### Source of AL fibrils

AL amyloid fibrils were extracted from the heart of a woman (Supplementary Table 1), suffering from advanced heart failure due to AL amyloidosis^22,23^. First symptoms (dyspnea, fatigue) started 1 year before diagnosis of AL amyloidosis. A monoclonal plasma cell disorder (smoldering myeloma) was diagnosed at the same time as AL amyloidosis. Bone marrow cytology showed 19% plasma cells (<5% λ-positive in bone marrow histology) and interphase fluorescence in situ-hybridization analysis of CD138+ enriched plasma cells showed the t(11;14) translocation and the 13q14 deletion. The patient received 5 months of treatment with bortezomib and dexamethasone, and achieved a serological complete remission of the smoldering myeloma. Ten months later, free λ-LCs increased and treatment with lenalidomide and dexamethasone was started but stopped after 2 months due to cardiac decompensation. 1 month later high urgency listing was done and the transplantation was performed 2 months later. Informed consent was obtained from the patient for the analysis of the amyloid deposits.

The fibril extraction was performed from heart muscle tissue as described previously^22^. In brief, 250 mg of tissue were diced and washed five times with 0.5 mL Tris calcium buffer (20 mM Tris, 138 mM NaCl, 2 mM CaCl_2_, 0.1 % NaN_3_, pH 8.0). Each washing step consisted of gentle vortexing and centrifugation at 3100 × *g* for 1 min at 4 °C. The supernatant was discarded and the pellet was resuspended in 1 mL of freshly prepared 5 mg mL^−1^*Clostridium histolyticum* collagenase (Sigma) in Tris calcium buffer. After incubation overnight at 37 °C the tissue material was centrifuged at 3100 × *g* for 30 min at 4 °C. The retained pellet was resuspended in 0.5 mL buffer containing 20 mM Tris, 140 mM NaCl, 10 mM ethylenediaminetetraacetic acid, 0.1 % NaN_3_, pH 8.0, and subjected to 10 cycles of homogenization in fresh buffer and centrifugation for 5 min at 3100 × *g* at 4 °C. The remaining pellet was homogenized in 0.5 mL ice cold water, centrifuged for 5 min at 3100 × *g* at 4 °C and the fibril-containing supernatant was analyzed. The study was approved by the ethical committees of the University of Heidelberg (123/2006) and of Ulm University (210/13)

### Measurement of the persistence length

Patient-derived fibrils were dried onto a carbon-coated grid and negatively stained with uranyl acetate as described previously^22^. Images were taken at 120 kV using a JEM-1400Plus microscopy (Jeol) equipped with a TemCam-F216 camera (TVIPS). The contour length *L* and end-to-end distance *D* of 124 well-resolved fibrils was determined using the program Fiji (http://fiji.sc). The plot of the squared end-to-end distance was fit with Eq. (1) to obtain the persistence length *P*:

1 $$\left( {D^2} \right) = 4PL \cdot \left[ {1 - \frac{{2P}}{L}\left( {1 - e^{\left( { - L/2P} \right)}} \right)} \right]$$

The persistence length can be converted into the bending rigidity *B* as described by:

2 $$B = P \cdot k_{\mathrm {B}} \cdot T$$

In this equation, *k*_B_ refers to the Boltzmann constant and *T* to the temperature (300 K).

### Cryo-EM

A 3.5 μL aliquot was applied to a glow-discharged holey carbon-coated grid (C-flat 1.2/1.3 400 mesh) blotted from the back side after an incubation time of 4 s at a humidity of >80% and plunge-frozen in liquid ethane using a Cryoplunge 3 System (Gatan). For image acquisition a K2-Summit detector (Gatan) in counting mode on a Titan Krios transmission electron microscope (Thermo Fisher Scientific) at 300 kV was used, using a Gatan imaging filter with a 20 eV slit. Table 1 lists the data acquisition parameters. The raw data have been deposited at https://www.ebi.ac.uk/pdbe/emdb/ with the accession code EMPIAR-10245.

### Helical reconstruction

The raw data movie frames were gain-corrected with IMOD^40^ and aligned, motion-corrected and dose-weighted using MOTIONCOR2^41^. Gctf^42^ was used to estimate the contrast transfer function from the aligned and motion-corrected images. RELION 2.1^43^ was used for the helical reconstruction of the fibril density. After manual selection of the fibrils from the aligned, motion-corrected micrographs, segments were extracted with a box size of ~333 Å and an inter-box distance of ~9% of the box length. Reference-free 2D classification with a regularization value of *T* = 3 produced class averages showing the helical repeat along the fibril axis. Class averages were selected based on a manual arrangement into a fibril structure. From the selected class averages, 200 randomly picked particles per class were used to generate an initial 3D model using the Stochastic Gradient Descent algorithm implemented in RELION. These initial models were low-pass filtered to 60 Å and used to generate a single-fibril model of two selected fibrils with clearly visible cross-overs. The single-fibril model showed the general shape of the fibril, and was then used as an initial model for 3D classification with *K* = 4. Two out of four classes were selected. The ~30k particles from these two classes were then used for a second 3D classification run with *T* = 3 using as a reference the model showing most features from the initial classification. This second classification produced a model showing the peptide backbone in the cross-section. Increasing the *T*-value to 20 and performing a third 3D-classification yielded a model showing a clear β-strand separation and some side-chain densities. Repeating the 3D-classification several times while increasing the *T*-value and decreasing the angular sampling rate further improved the model. Auto-refinement and post-processing with a soft-edged mask and an estimated map sharpening B-factor of −119.6 Å^2^ yielded the final model with a twist of 0.58° and 4.8 Å helical rise which are in good agreement with measurements of the cross-over distances, layer-line profiles of the 2D class averages and power spectra of the raw data. 3D classification and auto-refine processes used a central part of 10% of the intermediate reconstruction^43^ (except the initial classification with *K* = 4 which used 30%). Resolution estimates were obtained from the FSC at 0.143 between two independently refined half-maps.

### Model building and refinement

An initial model was built into a B-factor sharpened map (phenix.auto_sharpen)^44^ by the ARP/wARP EM module^45^ followed by manually rebuilding in Coot^46^. The resulting model was refined by real space refinement using phenix.real_space_refine^47^ with NCS and secondary structure restraints.

### Sequence analysis

V-germline, J-germline, and C-germline segments and CDRs were identified by http://www.vbase2.org/ database search^48^, BLAT search on UCSC (http://genome-euro.ucsc.edu/)^49^, and BLAST/BLAT search on ENSEMBL (http://www.ensembl.org/Multi/Tools/Blast?db = core)^50^ of the disposed fibril protein and its respective cDNA sequence. From these databases, cDNA and peptide sequences from the best candidates for each segment (fit > 80%) were retrieved and analyzed segment-wise for their fit to the disposed fibril protein using “MEGA Molecular Evolutionary Genetics Analysis” software and the multiple sequencing alignment tool “MUSCLE”^51^. Genetic distances were calculated by Maximum Composite Likelihood to identify the most probable V-segment, J-segment, and C-segment family which were then simultaneously aligned to the fibril protein and its respective cDNA sequence in order to gain a full length picture of sequence variation by MEGA and Clustal Omega (https://www.ebi.ac.uk/Tools/msa/clustalo/)^52^. The hydropathy score for each amino acid from residue Gly15 to Thr105 was assigned according to the Kyte–Doolittle scale of hydropathy^53^. The amyloid score for each residue was calculated based on the prediction of amyloid-prone regions using the programs WALTZ^54^, TANGO^55^, AmylPred^56^, Foldamyloid^57^, and Aggrescan^58^. An amyloid score of 0 means that none of the five programs identifies this residue as amyloidogenic. An amyloid score of 5 means that all five programs suggest this residue to be aggregation prone (Supplementary Table 3). WALTZ: values above 0.00 are considered as hits. TANGO: Beta sheet aggregation values above 0.00 are considered as hits. Fold amyloid: Amino acid residues having values above 21.4 for five consecutive residues are considered as hits. Aggrescan: Amino acid residues having value greater than −0.02 are considered as hot spots. AmylPred: Sequence regions predicted as hits by the Consensus method.

### Protein structure representation

Representations of reconstructed densities and refined models were created with UCSF Chimera^59^. A native LC structure was reproduced from protein data base (PDB) entry 1BJM^60^ based on its sequential similarity to our fibril protein and the presence of an *IGLV1-44* germ line segment.

### Reporting summary

Further information on experimental design is available in the Nature Research Reporting Summary linked to this article.

## Supplementary information

Supplementary Information

Reporting Summary

Source Data

## Data Availability

The reconstructed cryo-EM map was deposited in the Electron Microscopy Data Bank with the accession code EMD-4452. The coordinates of the fitted atomic model were deposited in the PDB under the accession code 6IC3. The Cryo-EM data were deposited on EMPIAR with the accession code EMPIAR-10245. The datasets and materials used during the current study are available from the corresponding author on reasonable request. The data underlying the Supplementary Figures 1 and 2 are provided as a Source Data File.

## References

[1] Ramirez-Alvarado M (2018). Systemic misfolding of immunoglobulins in the test tube and in the cell. Faseb J..

[2] van der Kant R (2017). Prediction and reduction of the aggregation of monoclonal antibodies. J. Mol. Biol..

[3] Gertz MA (2016). Immunoglobulin light chain amyloidosis: 2016 update on diagnosis, prognosis, and treatment. Am. J. Hematol..

[4] Quock TP, Yan T, Chang E, Guthrie S, Broder MS (2018). Epidemiology of AL amyloidosis: a real-world study using US claims data. Blood Adv..

[5] Buxbaum JN, Chuba JV, Hellman GC, Solomon A, Gallo GR (1990). Monoclonal immunoglobulin deposition disease: light chain and light and heavy chain deposition diseases and their relation to light chain amyloidosis. Clinical features, immunopathology, and molecular analysis. Ann. Intern. Med..

[6] Nienhuis HLA, Bijzet J, Hazenberg BPC (2016). The prevalence and management of systemic amyloidosis in western countries. Kidney Dis..

[7] Perfetti V (2012). The repertoire of λ light chains causing predominant amyloid heart involvement and identification of a preferentially involved germline gene, IGLV1-44. Blood.

[8] Wechalekar AD (2013). A European collaborative study of treatment outcomes in 346 patients with cardiac stage III AL amyloidosis. Blood.

[9] Merlini G, Comenzo RL, Seldin DC, Wechalekar A, Gertz MA (2014). Immunoglobulin light chain amyloidosis. Expert Rev. Gastroent..

[10] Schönland SO (2012). Immunohistochemistry in the classification of systemic forms of amyloidosis: a systematic investigation of 117 patients. Blood.

[11] Merlini G, Bellotti V (2003). Molecular mechanisms of amyloidosis. N. Engl. J. Med..

[12] Hurle MR, Helms LR, Li L, Chan W, Wetzel R (1994). A role for destabilizing amino acid replacements in light-chain amyloidosis. Proc. Natl Acad. Sci. USA.

[13] Blancas-Mejía LM, Ramirez-Alvarado M (2013). Systemic amyloidoses. Annu. Rev. Biochem..

[14] McWilliams-Koeppen, H. P. et al. Light chain amyloid fibrils cause metabolic dysfunction in human cardiomyocytes. *PLoS ONE* **9**, e0137716 (2015). 10.1371/journal.pone.0137716 PMC4579077 26393799

[15] Nokwe CN (2016). A Stable mutant predisposes anti-body domains to amyloid formation through specific non-native interactions. J. Mol. Biol..

[16] Comenzo RL, Zhang Y, Martinez C, Osman K, Herrera GA (2001). The tropism of organ involvement in primary systemic amyloidosis: contributions of Ig VL germ line gene use and clonal plasma cell burden. Blood.

[17] Abraham RS (2003). Immunoglobulin light chain variable (V) region genes influence clinical presentation and outcome in light chain-associated amyloidosis (AL). Blood.

[18] Kourelis TV (2017). Clarifying immunoglobulin gene usage in systemic and localized immunoglobulin light-chain amyloidosis by mass spectrometry. Blood.

[19] Pepys MB (2006). Amyloidosis. Annu. Rev. Med..

[20] Chiti F, Dobson CM (2017). Protein misfolding, amyloid formation, and human disease: a summary of progress over the last decade. Annu. Rev. Biochem..

[21] Sunde M (1997). Common core structure of amyloid fibrils by synchrotron X-ray diffraction. J. Mol. Biol..

[22] Annamalai K (2016). Polymorphism of amyloid fibrils in vivo. Angew. Chem. Int. Ed..

[23] Annamalai K (2017). Common fibril structures imply systemically conserved protein misfolding pathways in vivo. Angew. Chem. Int. Ed..

[24] Fitzpatrick AWP (2017). Cryo-EM structures of tau filaments from Alzheimer’s disease. Nature.

[25] Falcon B (2018). Structures of filaments from Pick’s disease reveal a novel tau protein fold. Nature.

[26] Gremer L (2017). Fibril structure of amyloid-β(1–42) by cryo-electron microscopy. Science.

[27] Guerrero-Ferreira, R. et al. Cryo-EM structure of alpha-synuclein fibrils. *eLife* **7**, e36402 (2018). 10.7554/eLife.36402 PMC6092118 29969391

[28] Liberta, F. et al. Cryo-EM structure of an amyloid fibril from systemic amyloidosis. Preprint at *bioRxiv* 10.1101/357129 (2018).

[29] Swuec, P. et al. Cryo-EM structure of cardiac amyloid fibrils from an immunoglobulin light chain (AL) amyloidosis patient. Preprint at *bioRxiv*10.1101/444901 (2018). 10.1038/s41467-019-09133-w PMC6427027 30894521

[30] Schmidt A, Annamalai K, Schmidt M, Grigorieff N, Fändrich M (2016). Cryo-EM reveals the steric zipper structure of a light chain-derived amyloid fibril. Proc. Natl Acad. Sci. USA.

[31] Blancas-Mejia LM (2018). Immunoglobulin light chain amyloid aggregation. Chem. Commun..

[32] del Pozo-Yauner L (2014). The N-terminal strand modulates immunoglobulin light chain fibrillogenesis. Biochem. Biophys. Res. Commun..

[33] Piehl W (2017). Immunoglobulin light chains form an extensive and highly ordered fibril involving the N- and C-termini. ACS Omega.

[34] Fersht, A. *Structure and Mechanism in Protein Science* (W.H. Freeman and Company, New York, 1999).

[35] Choi B, Kim T, Lee SW, Eom K (2016). Nanomechanical characterization of amyloid fibrils using single-molecule experiments and computational simulations. J. Nanomater..

[36] White HE (2009). Globular tetramers of β2-microglobulin assemble into elaborate amyloid fibrils. J. Mol. Biol..

[37] Brumshtein B (2014). Formation of amyloid fibers by monomeric light chain variable domains. J. Biol. Chem..

[38] Brumshtein, B. et al. Identification of two principal amyloid-driving segments in variable domains of Ig light chains in systemic light chain amyloidosis. *J. Biol. Chem*. **293**, 19659–19671 (2018). 10.1074/jbc.RA118.004142 PMC6314132 30355736

[39] Hora, M. et al. MAK33 antibody light chain amyloid fibrils are similar to oligomeric precursors *PLoS ONE* **7**, e0181799 (2017). 10.1371/journal.pone.0181799 PMC5528828 28746363

[40] Kremer JR, Mastronarde DN, McIntosh JR (1996). Computer visualization of three-dimensional image data using IMOD. J. Struct. Biol..

[41] Zheng SQ (2017). MotionCor2: anisotropic correction of beam-induced motion for improved cryo-electron microscopy. Nat. Methods.

[42] Zhang K (2016). Gctf: Real-time CTF determination and correction. J. Struct. Biol..

[43] He S, Scheres SHW (2017). Helical reconstruction in RELION. J. Struct. Biol..

[44] Afonine PV (2018). New tools for the analysis and validation of cryo-EM maps and atomic models. Acta Crystallogr. D.

[45] Langer G, Cohen SX, Lamzin VS, Perrakis A (2008). Automated macromolecular model building for X-ray crystallography using ARP/wARP version 7. Nat. Protoc..

[46] Emsley P, Lohkamp B, Scott WG, Cowtan K (2010). Features and development of coot. Acta Crystallogr. D.

[47] Afonine PV (2018). Real-space refinement in PHENIX for cryo-EM and crystallography. Acta Crystallogr. D.

[48] Retter I, Althaus HH, Munch R, Muller W (2005). VBASE2, an integrative V gene database. Nucleic Acids Res..

[49] Kent WJ (2002). BLAT--the BLAST-like alignment tool. Genome Res..

[50] Altschul SF, Gish W, Miller W, Myers EW, Lipman DJ (1990). Basic local alignment search tool. J. Mol. Biol..

[51] Tamura K, Stecher G, Peterson D, Filipski A, Kumar S (2013). MEGA6: Molecular Evolutionary Genetics Analysis version 6.0. Mol. Biol. Evol..

[52] Sievers F (2011). Fast, scalable generation of high-quality protein multiple sequence alignments using Clustal Omega. Mol. Syst. Biol..

[53] Kyte J, Doolittle RF (1982). A simple method for displaying the hydropathic character of a protein. J. Mol. Biol..

[54] Maurer-Stroh S (2010). Exploring the sequence determinants of amyloid structure using position-specific scoring matrices. Nat. Methods.

[55] Fernandez-Escamilla AM, Rousseau F, Schymkowitz J, Serrano L (2004). Prediction of sequence-dependent and mutational effects on the aggregation of peptides and proteins. Nat. Biotechnol..

[56] Frousios KK, Iconomidou VA, Karletidi CM, Hamodrakas SJ (2009). Amyloidogenic determinants are usually not buried. BMC Struct. Biol..

[57] Garbuzynskiy SO, Lobanov MY, Galzitskaya OV (2010). FoldAmyloid: a method of prediction of amyloidogenic regions from protein sequence. Bioinformatics.

[58] Conchillo-Solé O (2007). AGGRESCAN: a server for the prediction and evaluation of “hot spots” of aggregation in polypeptides. BMC Bioinforma..

[59] Pettersen EF (2004). UCSF Chimera—a visualization system for exploratory research and analysis. J. Comput. Chem..

[60] Huang DB, Ainsworth CF, Stevens FJ, Schiffer M (1996). Three quaternary structures for a single protein. Proc. Natl Acad. Sci. USA.

