# Supplementary material for: Cryo-EM structure of a light chain-derived amyloid fibril from a patient with systemic AL amyloidosis
Source: Nat Commun. 2019 Mar 20;10:1103. doi: 10.1038/s41467-019-09032-0 (PMC6427026; doi:10.1038/s41467-019-09032-0)
Supplement: Supplementary file 1 — Supplementary Information [file 41467_2019_9032_MOESM1_ESM.pdf]

Supplementary Information

Cryo-EM structure of a light chain-derived amyloid fibril from a patient with systemic AL amyloidosis

L. Radamaker et al.

Supplementary Figure 1

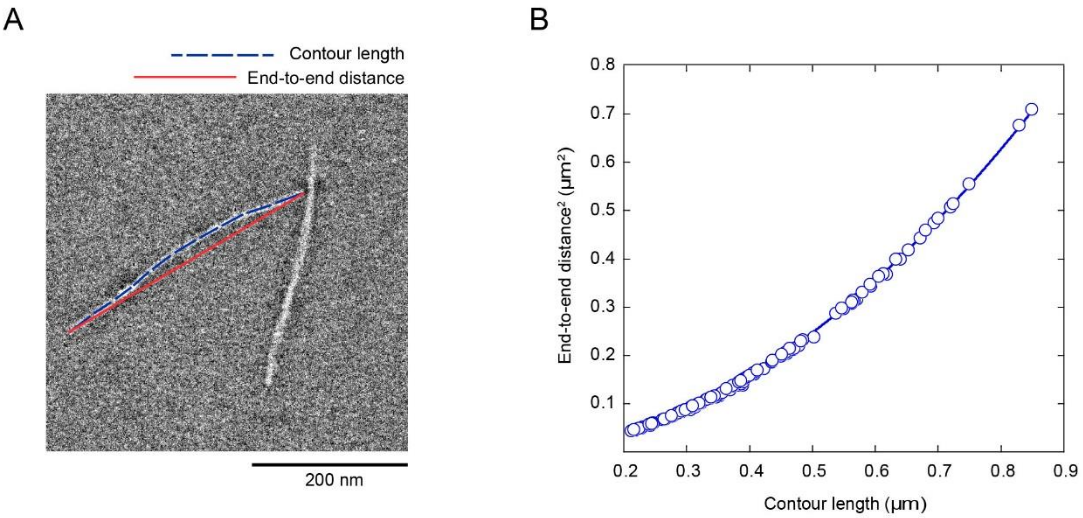

Quantification of the fibril bending rigidity

(a) Representative negatively stained TEM image of the analyzed fibrils showing the contour length and the end-to-end distance. (b) Plot of the squared end-to-end distance versus the contour length for 124 fibrils, fitted with a formula as described in the methods to obtain the fibril persistence length. The fit produced a value of 6.7 ± 0.5 µm for the persistence length and of 2.78 ± 0.21 10^{-26} Nm^2 for the bending rigidity (R^2 = 0.9995, errors refer to standard deviation).

Supplementary Figure 2

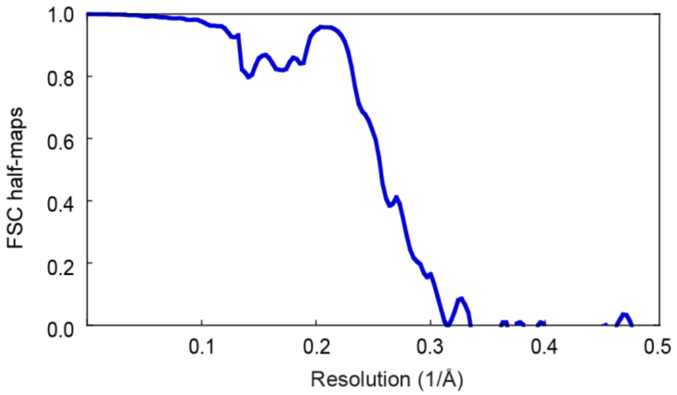

FSC curve of the reconstructed density

Supplementary Figure 3

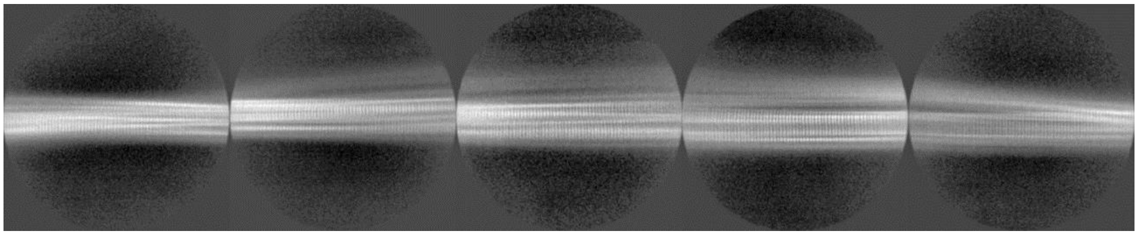

2D class averages

Selection of 2D class averages showing five classes which roughly cover one cross-over distance.

Supplementary Figure 4

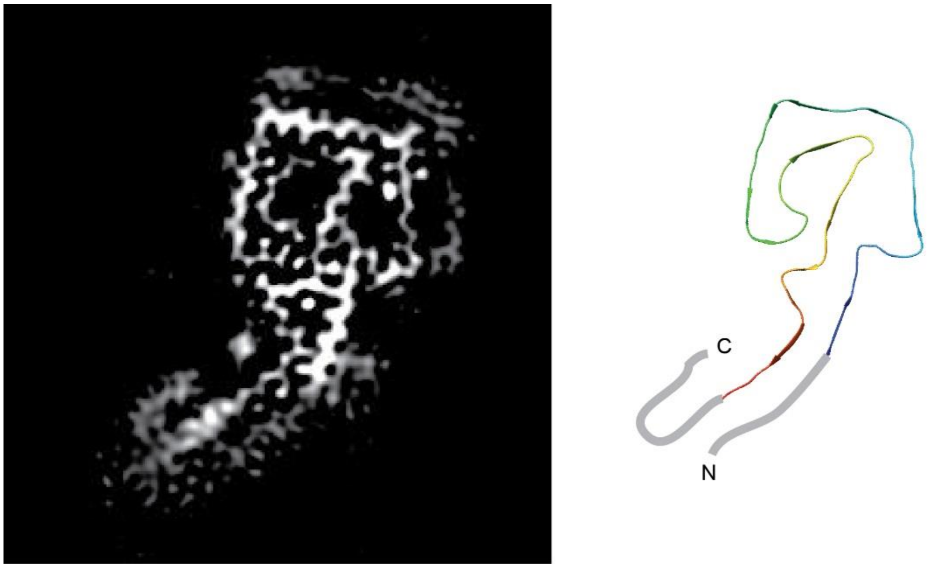

Presence of ordered and disordered regions in the fibril structure

Left: A 29 Å thick cross-sectional slice of the unmasked 3D map of the AL fibril. Right: Ribbon diagrams of one fibril molecule with a schematic drawing of the disordered C-terminus (grey). The drawing of the C-terminus corresponds to some of the diffuse density in the 3D map. Additional diffuse density of uncertain origin can be seen, similar to other *ex vivo* fibrils[1,2,3].

Supplementary Figure 5

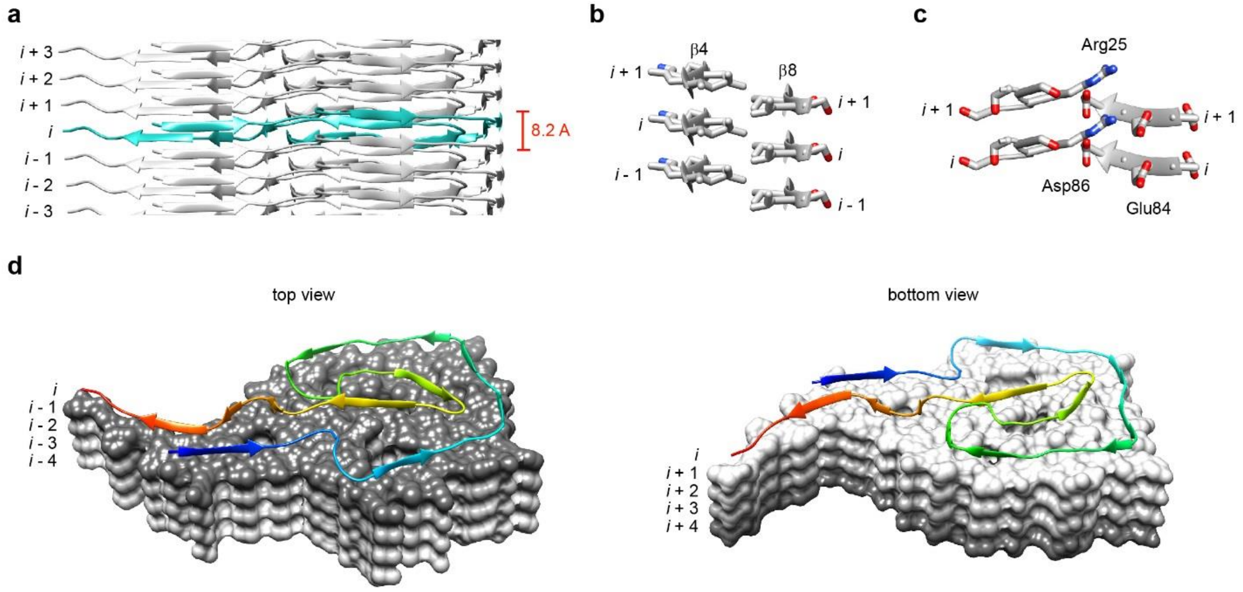

Intermolecular interactions along the fibril axis

(a) The height change of the fibril protein (8.2 Å) was determined between the Cα atom of Asn28 (highest point) and the Cα of Ala72 (lowest point). (b) Staggering of sheet β4 relative to sheet β8. Sheet β4 from layer *i* interacts with sheets β8 in layers in layers *i* and *i* + 1. (c) Interaction of Arg 25 from layer *i* with Glu84 and Asp86 in layer *i* + 1. (d) Different structural topology of the two fibril tips (left/right). Four layers are shown in gray scale (spacefill). The terminal layer is shown as a ribbon diagram in rainbow color from N (blue) to C (red).

Supplementary Table 1

Patient characteristics

**Parameter**	**At AL diagnosis**	**At heart transplantation**
Karnofsky Index / New York Heart Association cardiac stage	80% / III	70% / III-IV
Difference free LC value	137 mg/l	40 mg/l
Concentration of λ-LC in the urine	36 mg/d	Not determined
N-terminal pro brain natriuretic peptide concentration in the plasma	8,300 ng/l	19,800 ng/l
High sensitive troponin T concentration in the plasma	53 pg/ml	136 pg/ml
Cardiac stage	III A	III B
Septum thickness	18 mm	19 mm
Estimated glomerular filtration rate	94 ml/min	56 ml/min

Supplementary Table 2

Modeling parameters

Model resolution (Å)	
FSC threshold 0.143	3.3
FSC threshold 0.5	3.9
Model resolution range (Å)	330-3.3
Map sharpening *B* factor (Å^2)	-119.6
Model composition	
Non-hydrogen atoms	5472
Protein residues	728
Ligands	-
B factors (Å^2)	
Protein	111.43
Ligand	-
R.m.s. deviations	
Bond lengths (Å)	0.010
Bond angles (°)	1.257
Validation	
MolProbity score	2.29
Clashscore	9.22
Poor rotamers (%)	1.4
Ramachandran plot	
Favored (%)	83.1
Allowed (%)	16.9
Disallowed (%)	0

Supplementary Table 3

Analysis of the aggregation score for residues Val3-Ser118. Please refer to the Methods section for details. Hits are presented in bold print, and for AmylPred as +

Sequence	WALTZ	TANGO	Fold amyloid	Aggrescan	AmylPred	Score
V	0.00	0.00	23.00	-0.10	–	0
L	0.00	0.00	22.10	-0.10	–	0
T	0.00	0.00	21.20	-0.10	–	0
Q	0.00	0.00	19.90	0.09	–	0
P	0.00	0.00	18.40	-0.14	–	0
P	0.00	0.00	18.40	-0.38	–	0
S	0.00	0.00	18.20	-0.44	–	0
A	0.00	0.00	18.20	-0.28	–	0
S	0.00	0.00	18.60	-0.28	–	0
G	0.00	0.00	18.50	-0.31	–	0
T	0.00	0.00	17.90	-0.45	–	0
P	0.00	0.00	18.10	-0.62	–	0
G	0.00	0.00	18.90	-0.35	–	0
Q	**87.96**	0.00	19.70	-0.30	–	1
R	**87.96**	0.00	20.20	**-0.01**	–	2
V	**87.96**	0.00	21.90	**-0.01**	–	2
T	**87.96**	0.00	21.70	**0.16**	–	2
I	**87.96**	0.00	22.20	**0.29**	–	2
S	**87.96**	0.00	21.10	**0.39**	–	2
C	0.00	0.00	20.50	**-0.01**	–	1
S	0.00	0.00	19.60	-0.03	–	0
G	0.00	0.00	19.60	-0.34	–	0
R	**80.60**	0.00	18.50	-0.48	–	1
S	**80.60**	0.00	18.60	-0.31	–	1
S	**80.60**	0.00	20.30	-0.34	–	1
N	**80.60**	0.00	19.50	-0.44	–	1
I	**80.60**	0.00	20.10	-0.45	–	1
G	**80.60**	0.00	20.20	-0.21	–	1
R	**80.60**	0.00	21.50	**0.06**	–	2
N	**91.97**	0.00	21.20	**0.11**	–	2
L	**91.97**	0.00	21.30	**0.00**	–	2
V	**91.97**	0.00	**22.80**	**0.24**	–	3
K	**91.97**	0.00	**24.30**	**0.24**	–	3
W	**79.93**	0.00	**23.00**	**0.25**	–	3
Y	**79.93**	0.00	**22.10**	**0.31**	–	3

Q	**79.93**	0.00	**24.00**	**0.03**	–	3
Q	**79.93**	0.00	**21.80**	**0.09**	–	3
F	**79.93**	0.00	20.00	-0.08	–	1
P	**79.93**	0.00	20.20	-0.25	–	1
G	0.00	0.00	20.30	-0.13	–	0
T	0.00	0.00	18.30	-0.08	–	0
A	0.00	0.00	18.40	-0.14	–	0
P	**79.93**	0.00	20.00	**0.11**	–	2
K	**97.99**	0.00	21.10	**0.45**	–	2
L	**87.29**	**27.12**	22.30	**0.63**	+	4
L	**87.29**	**27.30**	24.00	**0.60**	+	4
I	**87.29**	**27.30**	24.10	**0.46**	+	4
Y	**87.29**	**27.30**	22.70	**0.33**	+	4
S	**87.29**	**27.30**	21.10	-0.04	+	3
N	**87.29**	**12.36**	19.90	-0.42	–	2
D	0.00	0.00	18.90	-0.73	–	0
Q	0.00	0.00	18.70	-0.93	–	0
R	0.00	0.00	18.70	-0.97	–	0
P	0.00	0.00	18.60	-0.55	–	0
S	0.00	0.00	19.50	-0.34	–	0
G	0.00	0.00	18.80	-0.43	–	0
V	0.00	0.00	18.80	-0.43	–	0
P	0.00	0.00	19.40	-0.13	–	0
D	0.00	0.00	21.40	-0.13	–	0
R	0.00	0.00	20.20	-0.13	–	0
F	0.00	0.00	20.20	-0.40	–	0
S	0.00	0.00	20.30	-0.48	–	0
G	0.00	0.00	19.70	-0.26	–	0
S	0.00	0.00	17.90	-0.16	–	0
K	0.00	0.00	17.70	-0.44	–	0
S	0.00	0.00	18.20	-0.44	–	0
G	0.00	0.00	18.20	-0.36	–	0
T	0.00	0.00	18.60	-0.36	–	0
S	0.00	0.00	18.60	-0.03	–	0
A	0.00	**0.40**	20.30	**0.00**	–	2
S	0.00	**0.40**	20.30	**0.31**	–	2
L	0.00	**0.64**	21.50	**0.29**	–	2
A	0.00	**0.64**	21.10	**0.25**	–	2
V	0.00	**0.64**	20.90	**0.46**	–	2
S	0.00	**0.24**	20.90	**0.32**	–	2
G	0.00	**0.24**	20.80	**0.08**	–	2

L	0.00	**0.24**	19.60	-0.11	–	1
Q	0.00	0.00	19.50	-0.60	–	0
S	0.00	0.00	19.50	-0.76	–	0
E	0.00	0.00	18.00	-0.69	–	0
D	0.00	0.00	18.10	-1.15	–	0
E	0.00	0.00	17.90	-0.81	–	0
A	**79.93**	0.00	19.60	-0.60	–	1
D	**79.93**	0.00	21.30	-0.31	+	2
Y	**79.93**	**0.39**	**22.50**	-0.06	+	4
Y	**80.60**	**0.39**	**22.50**	0.14	+	4
C	**80.60**	**0.39**	**23.00**	0.29	+	4
A	**80.60**	**0.39**	**23.50**	0.29	+	4
A	**80.60**	**0.39**	**21.80**	0.12	+	4
W	**79.93**	**0.39**	21.10	-0.07	–	2
D	**79.93**	0.00	21.10	**0.05**	–	2
A	**79.93**	**12.73**	22.20	-0.14	–	2
T	**88.63**	**28.85**	20.20	-0.14	–	2
L	**88.63**	**11.30**	20.70	-0.14	+	3
N	**93.98**	**12.67**	**22.40**	**0.35**	+	5
A	**93.98**	**15.72**	**23.20**	**0.61**	+	5
W	**93.98**	**16.02**	**23.60**	**0.56**	+	5
V	**93.98**	**16.02**	**23.30**	**0.28**	+	5
F	**93.98**	**15.35**	**22.80**	**0.39**	+	5
G	**93.98**	**43.39**	20.50	**0.37**	–	3
G	**93.98**	**0.55**	19.70	**0.09**	–	3
G	0.00	0.00	17.80	**0.06**	–	1
T	0.00	0.00	19.40	-0.21	–	0
K	0.00	0.00	20.00	**0.09**	–	1
L	**82.61**	**0.63**	21.30	**0.37**	–	3
T	**82.61**	**0.63**	22.40	**0.40**	–	3
V	**82.61**	**0.63**	22.50	**0.25**	–	3
L	**82.61**	**0.63**	21.30	**0.33**	–	3
S	**82.61**	**0.63**	20.80	**0.00**	–	3
Q	**82.61**	0.00	19.60	**0.02**	–	3
P	0.00	0.00	18.50	-0.21	–	0
K	0.00	0.00	18.80	-0.46	–	0
A	0.00	0.00	18.50	-0.46	–	0
A	0.00	0.00	18.60	-0.44	–	0
P	0.00	0.00	18.90	-0.44	–	0
S	0.00	0.00	18.50	-0.44	–	0

Supplementary References

[1] Fitzpatrick, A.W.P. *et al.* Cryo-EM structures of tau filaments from Alzheimer's disease. *Nature* **547**, 185-190 (2017)

[2] Liberta, F. *et al.* Cryo-EM structure of an amyloid fibril from systemic amyloidosis. Preprint at https://www.biorxiv.org/content/early/2018/06/29/357129 (2018)

[3] Swuec, P. *et al.* Cryo-EM structure of cardiac amyloid fibrils from an immunoglobulin light chain (AL) amyloidosis patient. Preprint at https://www.biorxiv.org/content/early/2018/10/17/444901 (2018)
